# Maternal nutrient restriction during late gestation reduces vigor and alters blood chemistry and hematology in neonatal beef calves

**DOI:** 10.1093/jas/skad342

**Published:** 2023-10-03

**Authors:** Lindsey G Wichman, Colby A Redifer, Allison M Meyer

**Affiliations:** Division of Animal Sciences, University of Missouri, Columbia, MO 65211, USA; Division of Animal Sciences, University of Missouri, Columbia, MO 65211, USA; Division of Animal Sciences, University of Missouri, Columbia, MO 65211, USA

**Keywords:** beef cattle, developmental programming, passive transfer, perinatal, pregnancy, undernutrition

## Abstract

Fall-calving primiparous beef females [body weight: 451 ± 28 (SD) kg; body condition score: 5.4 ± 0.7] were individually-fed either 100% (control; CON; *n* = 13) or 70% (nutrient restricted; NR; *n* = 13) of metabolizable energy and metabolizable protein requirements for maintenance, pregnancy, and growth from day 160 of gestation to parturition. Calves were reared naturally by their dams and monitored for latency times from birth to first sternal recumbency, attempt to stand, and stand; vigor scores were assigned at 2, 5, 10, and 20 min of age. Rectal temperatures and jugular blood were obtained at 0 (pre-suckling), 6, 12, 24, and 48 h of age, and blood chemistry, hematology, cortisol, and insulin were determined. Data were analyzed with fixed effects of late gestational nutritional plane (single data point) or nutritional plane, hour, and their interaction (data over time, repeated measures). Calving date was a fixed effect; calf sex was included when *P* < 0.25. We previously reported that late gestational nutritional plane did not affect gestation length or calf size at birth, but calving assistance and fetal malpresentation occurred more often in NR. Nutritional plane did not affect (*P =* 0.65) duration of parturition, but calves born to NR dams had slower times to attempt to stand (*P* = 0.09), slower times to stand (*P* = 0.02), and poorer 20 min vigor scores (*P* = 0.05). Serum immunoglobulin G and A concentrations at 48 h were greater (*P* ≤ 0.03) for NR calves. Rectal temperature of NR calves was less (*P* = 0.02) at 0 h, but greater (*P* = 0.04) at 24 h compared with CON. Circulating glucose, non-esterified fatty acids, triglycerides, cortisol, and insulin were not affected by nutritional plane (*P* ≥ 0.18). Total protein and globulin from 6 to 48 h were greater (*P* ≤ 0.02) in NR calves. Calves from NR dams had greater (*P* ≤ 0.08) gamma-glutamyl transferase at 6, 12, and 48 h. Serum aspartate aminotransferase was greater (*P* ≤ 0.07) from 0 to 24 h and creatine kinase was greater (*P* ≤ 0.04) from 6 to 24 h in NR calves. At 0 h, potassium was greater (*P* = 0.03) in NR calves. Calves born to CON had greater chloride (*P* = 0.08; main effect), sodium (*P* ≤ 0.09) from 0 to 48 h, and anion gap (*P* = 0.02) at 6 h. Hematocrit from 6 to 24 h and red blood cells and hemoglobin at 6 and 12 h were greater (*P* ≤ 0.09) in CON calves. These data indicate that nutrient restriction during late gestation resulted in less vigorous calves with more indicators of trauma in early life.

## Teaser

Beef calves born to heifers that were nutrient restricted during late gestation were less vigorous at birth and had more indicators of calving trauma in the first 48 h of life. Despite this, their energy status was minimally affected, and they achieved successful transfer of passive immunity.

## Introduction

Poor maternal nutrition during gestation can alter nutrient delivery to the fetus, ultimately impairing prenatal growth and development (Caton and Hess, 2010; Meyer et al., 2012) as well as pre-weaning health and survival (Cooke, 2019; Perry et al., 2019). Survival through the neonatal period is one of the greatest challenges to beef calves, and it depends on a successful transition to extrauterine life (Danijela, 2015). Following birth, calves must be vigorous to quickly stand and ingest colostrum for necessary nutrients and transfer of passive immunity (Weaver et al., 2000; Hammon et al., 2013). Despite this, few intensive studies have been conducted in beef cattle to determine effects of poor nutrition during pregnancy on neonatal calf vigor and metabolism.

During the last third of gestation, nutrient requirements of beef females increase rapidly to match the increase in demand for nutrients by the fetus (NASEM, 2016); however, they often may not meet these requirements due to poor forage quality or quantity (DelCurto et al., 2000; Caton and Hess, 2010). Additionally, primiparous dams likely have the greatest challenge while attempting to meet both fetal demands and their own growth requirements (Holland and Odde, 1992; Wu et al., 2006). We have previously reported that neonatal calves born to primiparous females have impaired metabolic status compared with calves born to multiparous females (Duncan et al., 2023), suggesting that calves born to first-parity dams are already at a disadvantage regardless of maternal nutrition. Overall, more data are needed to understand how poor nutrition during late gestation affects neonatal calf physiology and nutrient availability. Data reported here are from a large experiment (Redifer et al., 2023) to determine the effects of nutrient restriction in late pregnant first-parity beef females on prenatal and postnatal nutrient availability and utilization by calves. We hypothesized that late gestational nutrient restriction would impair vigor and metabolic status of neonatal beef calves. Our objectives were to determine the effects of maternal nutrient restriction during late gestation on neonatal calf vigor, passive transfer, maintenance of body temperature, blood chemistry, and hematology.

## Materials and Methods

The University of Missouri Animal Care and Use Committee approved animal care and use (Protocol #9877) in this study which took place at the University of Missouri Beef Research and Teaching Farm (Columbia, MO).

### Animal management and diets

Animal management and diets during pregnancy have been described by Redifer et al. (2023). Briefly, 26 single-sired fall-calving Hereford × Simmental-Angus crossbred beef heifers [initial body weight (**BW**) = 451 ± 28 (SD throughout methods) kg, initial body condition score (**BCS**) = 5.4 ± 0.7] bred by artificial insemination to a single Angus sire were allocated by BW, BCS, fetal sex, and expected calving date to 1 of 2 late gestational nutritional planes from day 160 of gestation to parturition. Control (**CON**; *n* = 13) heifers were individually-fed 100% of metabolizable energy (**ME**) and metabolizable protein (**MP**) requirements for maintenance, pregnancy, and growth, whereas nutrient restricted (**NR**; *n* = 13) heifers were individually-fed 70% of ME and MP requirements. Heifers were housed in 12 partially-covered 3.7 × 15.8 m pens (*n* = 2 to 3 per pen), penned by nutritional plane, and individually-fed via a Calan gate feeding system (Calan Broadbent Feeding System, American Calan, Northwood, NH).

Nutrient requirements were estimated using an expected calf birth weight of 34 kg and a projected maternal average daily gain of 0.36 kg/d. Metabolizable energy for maintenance was based on data for heifers in confinement (0.138 Mcal ME/kg non-gravid BW^0.75^; Freetly and Hales, personal communication). The equation used for ME for conceptus was published previously (Freetly et al., 2005). Equations from NASEM (2016) were utilized for ME for gain and MP for maintenance, conceptus, and gain. Nutrient requirements were adjusted weekly using the most recent dam BW (recorded every 21 d) and day of gestation.

From days 160 to 265 of gestation, diets were based on chopped sorghum sudan hay [1.74 Mcal ME/kg, 6.69% crude protein (**CP**), 72.0% neutral detergent fiber (**NDF**), 52.8% acid detergent fiber (**ADF**); dry matter (**DM**) basis]. From day 266 of gestation to parturition, diets were based on chopped endophyte-infected tall fescue-based hay (1.90 Mcal ME/kg, 7.22% CP, 65.1% NDF, 43.2% ADF; DM basis). Feeding poor quality forage allowed animals fed both nutritional planes to consume ad libitum hay without exceeding the ME and CP targets at any point. Using expected individual hay intakes (estimated from the previous week’s hay intake), heifers were supplemented daily with whole corn, dried distillers’ grains with solubles, and soyhull pellets to meet their assigned nutritional plane. Dams had ad libitum access to water and a trace mineralized salt block (Big 6 Mineral Salt, Compass Minerals America Inc., Overland Park, KS).

### Calving monitoring and data collection

Heifers remained in partially covered pens during the peripartum period. Beginning on day 274 of gestation, heifers were closely monitored 24 h per day by trained personnel to detect when heifers were in stage 2 of parturition (evident contractions, raised tail, or appearance of amniotic fluid or membranes) by walking down aisles located in the front and back of the pens at least once every 15 to 30 min. Once stage 2 was detected, heifers were continuously monitored to allow for all data collection. Overhead lighting in the barn and outdoor lighting allowed for continuous monitoring of animals throughout the night, and a handheld spotlight was used as necessary. Calving assistance was provided (CON *n* = 2; NR *n* = 4) in the pen, or the dam was moved to the chute for delivery assistance if the calf was presenting abnormally, there was a prolonged duration since the first appearance of fetal membranes, or if progress slowed during contractions. A calving difficulty score ranging from 1 to 5 was assigned, with 1 = no assistance, 2 = easy pull (by hand or with obstetrical chains), 3 = mechanically-assisted pull, 4 = abnormal presentation, and 5 = cesarean-section. No calving difficulty score 5 occurred.

Trained personnel recorded times for calving and vigor behaviors (Table 1) in real time. This allowed for the determination of duration of parturition, as well as calf vigor using both behavioral latencies and vigor scores. The estimated first appearance of fetal feet was used for 1 CON and 3 NR females based on the last time of monitoring prior to the appearance of feet, but actual times were used for all other measures. Behavioral latencies (Table 1) included time to sternal recumbency, time to first attempt to stand, and time to stand. A vigor score was also assigned to calves at 2, 5, 10, and 20 min of age on a scale of 1 to 5 [1 = very weak (lying flat on side), 2 = weak (flat on side with head up), 3 = active and vigorous (sternal), 4 = very active and vigorous (attempting to stand or has attempted to stand), and 5 = extremely active and vigorous (standing or has stood successfully)] as described in Duncan et al. (2023). The number of failed attempts to stand by each calf that were not caused by its dam was also recorded.

**Table 1.** Calving behavior and calf vigor definitions

Behavior	Definition
Appearance of fetal foot or feet	Visible expulsion of fetal foot or feet (may be in membranes), whether the foot or feet stay(s) visible or not
Birth	Expulsion of entire calf, including all 4 legs
Sternal recumbency	Calf lays on chest with head up
Attempt to stand	Calf supports weight on hind legs while balanced on the front knees for ≥5 s, without standing successfully (including standing for <5 s)
Standing	Calf stands on all 4 legs for ≥5 s
Measure	Definition
Duration of parturition, min	Time from first appearance of fetal foot or feet to birth
Time to sternal recumbency, min	Time from birth to first sternal recumbency
Time to first attempt to stand, min	Time from birth to first attempt to stand (not assigned if calf successfully stands first)
Time to stand, min	Time from birth to first standing
Number of failed attempts to stand	Number of attempts to stand that failed due to the calf falling, not including failed attempts caused by the dam (can be 0)

Following the successful standing of the calf, but prior to suckling, each calf was removed from the dam and processed. Each calf was given an ear tag for visual identification, had their umbilicus sprayed with chlorhexidine solution, and was administered a subcutaneous Bo-Se injection (5.5 mL/100 kg BW; Intervet/Merck Animal Health, Madison, NJ). The ­average calving date was September 16 ± 32.9 d, and there was no perinatal calf death loss.

### Neonatal blood collection and analyses

Calf jugular blood samples and rectal temperatures were collected at 0 (prior to suckling but after standing), 6, 12, 24, and 48 h of age (0.6 ± 0.3, 6.0 ± 0.2, 12.1 ± 0.2, 24.2 ± 0.2, 48.1 ± 0.3 h, respectively). Blood samples were collected into Vacutainer serum collection tubes containing no additives (2 tubes at 0 and 48 h, 1 tube at other sampling hours; 10 mL draw; Becton Dickinson, Franklin Lakes, NJ) and 1 Monoject plasma collection tube containing 0.10 mL of 15% K_3_EDTA (10 mL draw; Covidien, Mansfield, MA). Following collection, all tubes were inverted several times. Plasma tubes were immediately placed on ice, whereas serum tubes were allowed to clot for approximately 5 to 15 min prior to being placed on ice. Between 1 and 8 h after collection, all samples were centrifuged at 1,500 × *g* at 4°C for 30 min. Serum and plasma were transferred into multiple 2-mL microcentrifuge tubes and stored at −20 °C until analysis unless they were refrigerated for analysis as described below for blood chemistry.

To evaluate the transfer of passive immunity, 48-h calf serum was analyzed for immunoglobulin (**Ig**) G and A concentrations. These were analyzed by a colorimetric sandwich enzyme-linked immunosorbent assay (**ELISA**) using Bovine IgG and IgA ELISA Kits (Bethyl Laboratories, Inc., Montgomery, TX) following the manufacturer’s instructions and methods described in Redifer et al. (2023). Serum samples were thawed at 4 °C and diluted 1:250,000 for IgG and 1:7,500 for IgA in sterile polypropylene tubes using the dilution buffer provided with the kit. Samples were analyzed in duplicate, and pooled serum samples served as assay controls. The intraassay and interassay CV were 4.07% and 6.90% for serum IgG, respectively, and the intraassay CV was 3.04% for IgA (single plate).

Calf serum non-esterified fatty acids (**NEFA**) were determined as described by Niederecker et al. (2018), and plasma triglycerides were determined as described by Larson-Peine et al. (2022) at each sampling hour using commercially-available colorimetric assays. For each assay, samples were analyzed in duplicate, and pooled control samples were used. The intraassay and interassay CV were 3.83% and 4.46% for serum NEFA and 3.30% and 1.05% for plasma triglycerides, respectively.

A 1-mL serum aliquot and 1-mL whole blood aliquot (collected with Monoject plasma collection tube containing 0.10 mL of 15% K_3_EDTA; aliquoted after gentle tube inversion prior to centrifugation for plasma) from each sampling time were refrigerated and then transported on ice to the University of Missouri Veterinary Medical Diagnostic Clinical Pathology Laboratory for blood chemistry and complete blood cell analysis. Serum glucose, urea N, creatinine, globulin, albumin, total protein, sodium, potassium, chloride, phosphorus, calcium, magnesium, bicarbonate, anion gap, direct bilirubin, total bilirubin, aspartate aminotransferase (**AST**), gamma-glutamyl transferase (**GGT**), and creatine kinase (**CK**) concentrations were determined using a Beckman Coulter AU480 Chemistry Analyzer (Beckman Coulter, Inc., Brea, CA). White blood cells (**WBC**), red blood cells (**RBC**), hemoglobin, hematocrit, mean corpuscular volume (**MCV**), mean corpuscular hemoglobin (**MCH**), mean corpuscular hemoglobin concentration (**MCHC**), and platelet count were determined using a Sysmex XT-2000iV (Sysmex Nordic Aps Filial Sverige, Landskrona, Sweden). Internal quality control and verification of performance within specific CV were conducted daily. Upon delivery, samples were analyzed at the same time through the instrument’s completely automated process. Samples were analyzed within 24 h of collection.

Calf plasma insulin concentration at each sampling hour was analyzed by radioimmunoassay (**RIA**) in duplicate using a commercial kit (Human Insulin Specific RIA, EMD Millipore Corp., Billerica, MA) as previously described (Duncan et al., 2023). The intra-assay CV was 2.80% (single assay). Calf plasma cortisol concentration at each sampling time was analyzed using a commercial coated-tube RIA kit (ImmunChem Coated Tube Cortisol ^125^I RIA Kit, MP Biomedicals, Irvine, CA) in duplicate. Manufacturer instructions were followed, except samples were incubated with Cortisol-^125^I for 4 h at room temperature. The intra-assay CV was 1.09% (single assay).

### Statistical analyses

One heifer (CON) was removed from the study due to late gestational abortion, resulting in CON *n* = 12 and NR *n* = 13. Vigor score at 2 min and time to sternal recumbency were not included for 1 NR calf because it was born through the fence into an adjacent pen and required human intervention to be put back into its respective pen. The only vigor measures recorded for calves that had a calving difficulty score of 4 (3 NR) included time to stand and 20 min vigor score because calves were pulled in the chute, which disrupted earlier vigor behaviors. Vigor data from calves with a calving difficulty of 2 or 3 (2 CON and 1 NR) were included in the analysis because assistance was provided in the pen and no further intervention was provided following the birth of the calf. At least 4 of the 5 sampling times were present for all rectal temperature, blood chemistry, hormone, and hematology data (3 animals missing 1 time point for rectal temperature and 1 missed time point for hematology). The final *n* for each variable is given in tables and figures descriptions.

Calf vigor behavioral latencies and serum Ig were analyzed using the MIXED procedure in SAS 9.4 (SAS Institute Inc., Cary, NC) with late gestational nutritional plane as a fixed effect. For rectal temperature, blood chemistry, plasma hormone, and hematology data over time, late gestational nutritional plane, hour, and their interaction were considered fixed effects. These were considered repeated measures using the majority best-fit covariance structure (based on Akaike Information Criterion, Bayesian Information Criterion, and corrected Bayesian Information Criterion) specific for each variable (chosen from unstructured, compound symmetry, heterogeneous compound symmetry, autoregressive, and heterogeneous autoregressive). Calf vigor score data were analyzed using a cumulative logistic regression model with the GLIMMIX procedure in SAS 9.4 with late gestational nutritional plane as a fixed effect. For all measures, animal was considered the experimental unit, Julian date of calving was included as a fixed effect (to account for variation in ambient conditions at calving), and calf sex was included as a fixed effect when *P* < 0.25. Means were separated using least significant difference and considered different when *P* ≤ 0.05 and tendencies were considered when 0.05 < *P* ≤ 0.10. In the absence of interactions, main effects of nutritional plane are reported. Additionally, PROC CORR was used to determine the Pearson correlation coefficients for calf serum IgG with total protein, globulin, and GGT at 48 h, both within nutritional planes and for all data combined.

## Results

### Vigor and transfer of passive immunity

**Figure 1.**
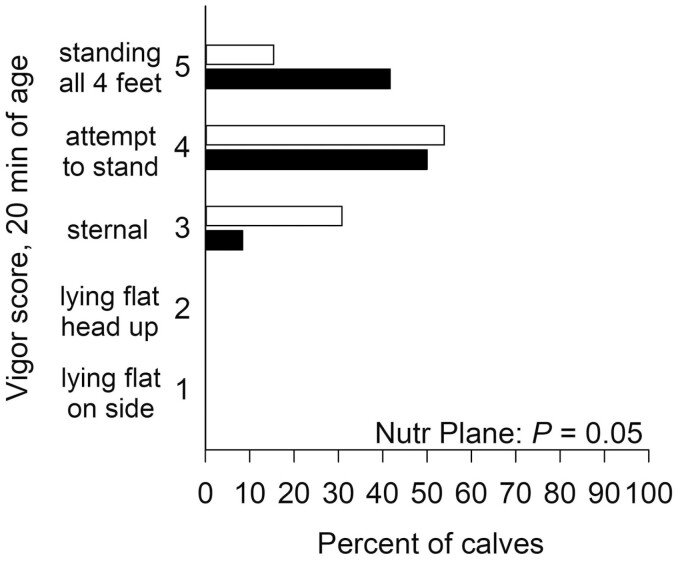
Effects of late gestational nutritional plane on frequency of calf vigor score at 20 min of age. Calves were born to primiparous beef females individually-fed 100% (Control; *n* = 12; solid bars, ■ ) or 70% (Nutrient Restricted; *n* = 13; open bars, □ ) of metabolizable energy and metabolizable protein requirements for maintenance, pregnancy, and growth from day 160 of gestation to parturition.

Duration of parturition was not affected (*P* = 0.65) by late gestational nutritional plane (CON: 40.6 ± 6.6 min vs. NR: 44.7 ± 6.3 min). Although there was no effect (*P* = 0.45) of late gestational nutritional plane on time to sternal recumbency, calves born to NR dams tended to attempt to stand 6.5 min (56%) slower (*P* = 0.09) and stood 14 min (67%) slower (*P* = 0.02) than calves born to CON dams (Table 2). Despite this, late gestational nutritional plane did not affect (*P* = 0.58) the number of failed attempts to stand. Calves born to NR dams were more likely to have a poorer (*P* = 0.05) vigor score at 20 min of age compared with CON calves (Figure 1), but late gestational nutritional plane did not affect (*P* ≥ 0.55) vigor scores at 2, 5, and 10 min of age (Supplementary Figure S1).

**Table 2.** Effects of late gestational nutritional plane of primiparous beef females on neonatal calf vigor and transfer of passive immunity^1^

Variable	Nutritional plane^2^		*P*-value
	Control	Nutrient restricted	
Calf vigor			
Time to sternal recumbency^3^, min	3.33 ± 0.47	2.78 ± 0.54	0.45
Time to attempt to stand^4^, min	11.7 ± 2.7	18.2 ± 2.7	0.09
Time to stand^5^, min	20.8 ± 4.1	34.8 ± 4.0	0.02
Number of failed attempts to stand^6^	5.33 ± 1.14	4.41 ± 1.25	0.58
48-h calf serum^5^			
Immunoglobulin G, mg/mL	29.9 ± 3.5	48.1 ± 3.4	0.001
Immunoglobulin A, mg/mL	1.46 ± 0.35	2.58 ± 0.34	0.03

^1^Mean ± SEM presented for measures.

^2^Primiparous dams were individually-fed either 100% (Control) or 70% (Nutrient Restricted) of metabolizable energy and metabolizable protein requirements for maintenance, pregnancy, and growth from day 160 of gestation to parturition.

^3^Control *n* = 12, Nutrient Restricted *n* = 9.

^4^Only included for calves with attempts to stand prior to standing. Control *n* = 10, Nutrient Restricted *n* = 10.

^5^Control *n* = 12, Nutrient Restricted *n* = 13.

^6^Control *n* = 12, Nutrient Restricted *n* = 10.

**Figure 2.**
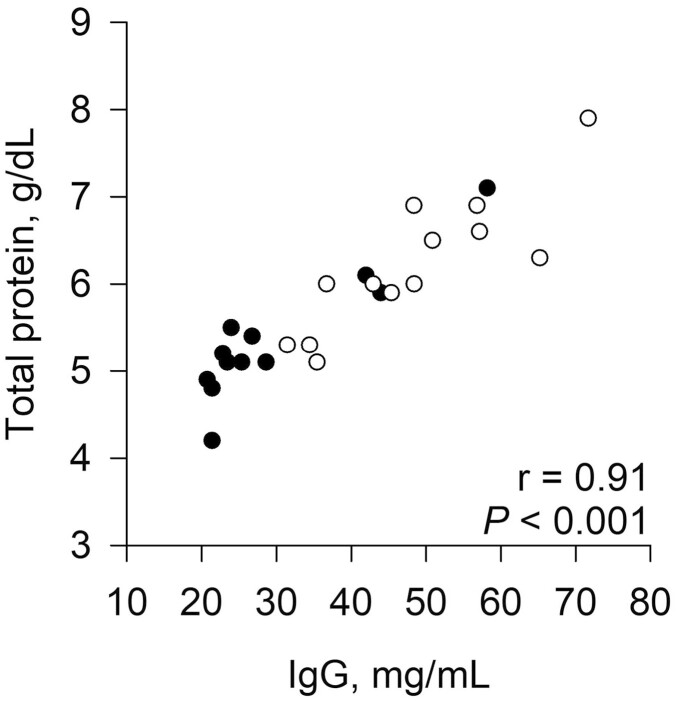
Relationship of serum immunoglobulin G (IgG) and total protein in calves at 48 h of age. Calves were born to primiparous beef females individually-fed 100% (Control; *n* = 12; solid circles, ● ) or 70% (Nutrient Restricted; *n* = 13; open circles, ○ ) of metabolizable energy and metabolizable protein requirements for maintenance, pregnancy, and growth from day 160 of gestation to parturition.

Calves born to NR dams had greater serum IgG (*P* = 0.001) and IgA (*P* = 0.03) at 48 h of age compared with calves born to CON (Table 2). At 48 h, serum IgG was very strongly correlated with serum total protein (*r* = 0.86 to 0.92, *P* < 0.001; Figure 2) and globulin (*r* = 0.87 to 0.96, *P* < 0.001) for both nutritional planes, and all data combined. When all data were combined, serum IgG was not correlated with GGT (*r* = 0.29, *P* = 0.16). Serum IgG tended to be moderately correlated (*r* = 0.54, *P* = 0.07) with GGT for calves born to CON, but there was no relationship (*r* = −0.08, *P* = 0.81) for NR.

### Rectal temperature and blood chemistry

**Figure 3.**
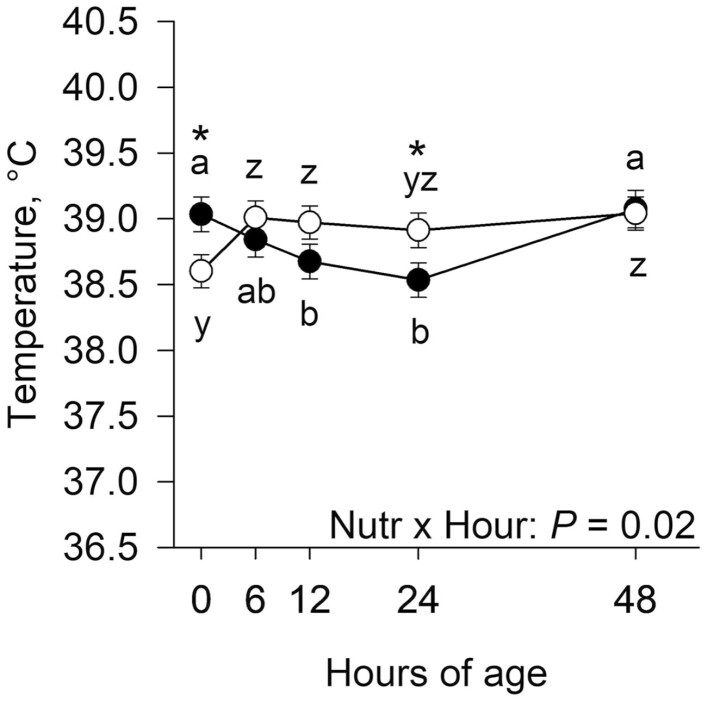
Effects of late gestational nutritional plane on neonatal calf rectal temperature. Calves were born to primiparous beef females individually-fed 100% (Control; *n* = 10 to 12; solid circles, ● ) or 70% (Nutrient Restricted; *n* = 12 to 13; open circles, ○ ) of metabolizable energy and metabolizable protein requirements for maintenance, pregnancy, and growth from day 160 of gestation to parturition. Least squares means ± SEM are presented. *Nutritional plane means within hour differ (*P* ≤ 0.05). ^a,b^Means differ (*P* ≤ 0.05) for Control calves across hours. ^y,z^Means differ (*P* ≤ 0.05) for Nutrient Restricted calves across hours.

The average Julian date of calving (261 ± 10 vs. 260 ± 9 for CON and NR, respectively; *P* = 0.93) and ambient temperature during the hour of calving (from on-farm weather station; 21.7 ± 2.1 vs. 20.2 ± 2.0 °C for CON and NR, respectively, *P* = 0.61) did not differ between late gestational nutritional planes. There was an interaction (*P* = 0.02) of late gestational nutritional plane × hour for neonatal calf rectal temperature (Figure 3). Calves born to CON dams had greater (*P* = 0.02) rectal temperature at 0 h of age; however, at 24 h postnatal, calves from NR dams had greater (*P* = 0.04) rectal temperature. Within CON calves, rectal temperatures at 0 and 48 h were greater (*P* ≤ 0.05) than at 12 and 24 h postnatal. Rectal temperature of NR calves increased (*P* = 0.01) from 0 to 6 h of age, but did not change (*P *≥ 0.45) from 6 to 48 h.

Neither the late gestational nutritional plane × hour interaction nor nutritional plane affected (*P* ≥ 0.18) neonatal calf serum glucose, serum NEFA, or plasma triglyceride concentrations (Supplementary Figure S2). Additionally, plasma cortisol and insulin were not affected (*P* ≥ 0.46) by the late gestational nutritional plane × hour interaction or nutritional plane (Supplementary Figure S3).

**Figure 4.**
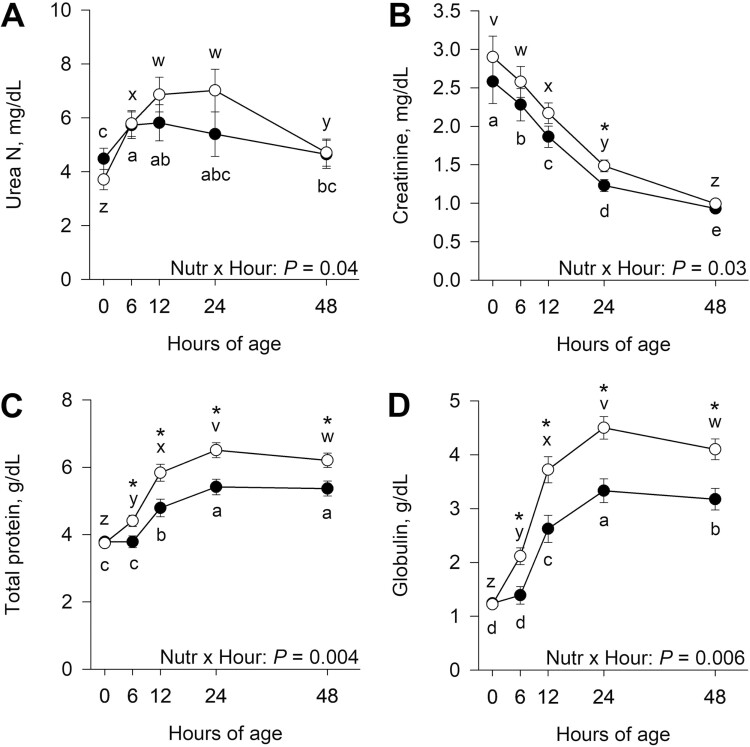
Effects of late gestational nutritional plane on neonatal calf serum urea N (Panel A), creatinine (Panel B), total protein (Panel C), and globulin (Panel D). Calves were born to primiparous beef females individually-fed 100% (Control; *n* = 12; solid circles, ● ) or 70% (Nutrient restricted; *n* = 13; open circles, ○ ) of metabolizable energy and metabolizable protein requirements for maintenance, pregnancy, and growth from day 160 of gestation to parturition. Least squares means ± SEM are presented. *Nutritional plane means within hour differ (*P* ≤ 0.05). ^a,b,c,d,e^Means differ (*P* ≤ 0.05) for Control calves across hours. ^v,w,x,y,z^Means differ (*P* ≤ 0.05) for Nutrient Restricted calves across hours.

There was an interaction (*P* = 0.04) of late gestational nutritional plane × hour for serum urea N concentrations (Figure 4A). Although urea N did not differ (*P* ≥ 0.17) between nutritional planes at any of the sampling time points, the pattern of change differed between nutritional planes. Within calves born to CON dams, serum urea N increased (*P* < 0.001) from 0 to 6 h of age and was less (*P* = 0.02) at 48 h than 6 h. Serum urea N increased (*P* < 0.001) from 0 to 12 h, but then decreased (*P* < 0.001) from 24 to 48 h postnatal in calves born to NR dams. There was also an interaction (*P* = 0.03) of late gestational nutritional plane × hour for serum creatinine, where NR calves had greater (*P* = 0.03) creatinine concentration at 24 h of age compared with CON calves (Figure 4B). Serum creatinine decreased (*P* ≤ 0.004) from 0 to 48 h of age in both CON and NR calves. There was an interaction (*P* ≤ 0.006) of late gestational nutritional plane × hour for serum total protein and globulin (Figure 4C and D). Calves born to NR dams had greater (*P* ≤ 0.02) serum total protein and globulin than CON at 6, 12, 24, and 48 h of age. Serum total protein increased (*P* < 0.001) from 6 to 24 h for both CON and NR. In calves born to NR dams, total protein also increased (*P* < 0.001) from 0 to 6 h and decreased (*P* < 0.001) from 24 to 48 h of age. Serum globulin had a similar pattern to total protein, increasing (*P* < 0.001) from 6 to 24 h of age and then decreasing (*P* ≤ 0.01) from 24 to 48 h in both CON and NR calves. Calves born to NR dams also had an increase (*P* < 0.001) in globulin from 0 to 6 h. There was no effect (*P *≥ 0.39) of the late gestational nutritional plane × hour interaction or nutritional plane on serum albumin (data not shown).

**Figure 5.**
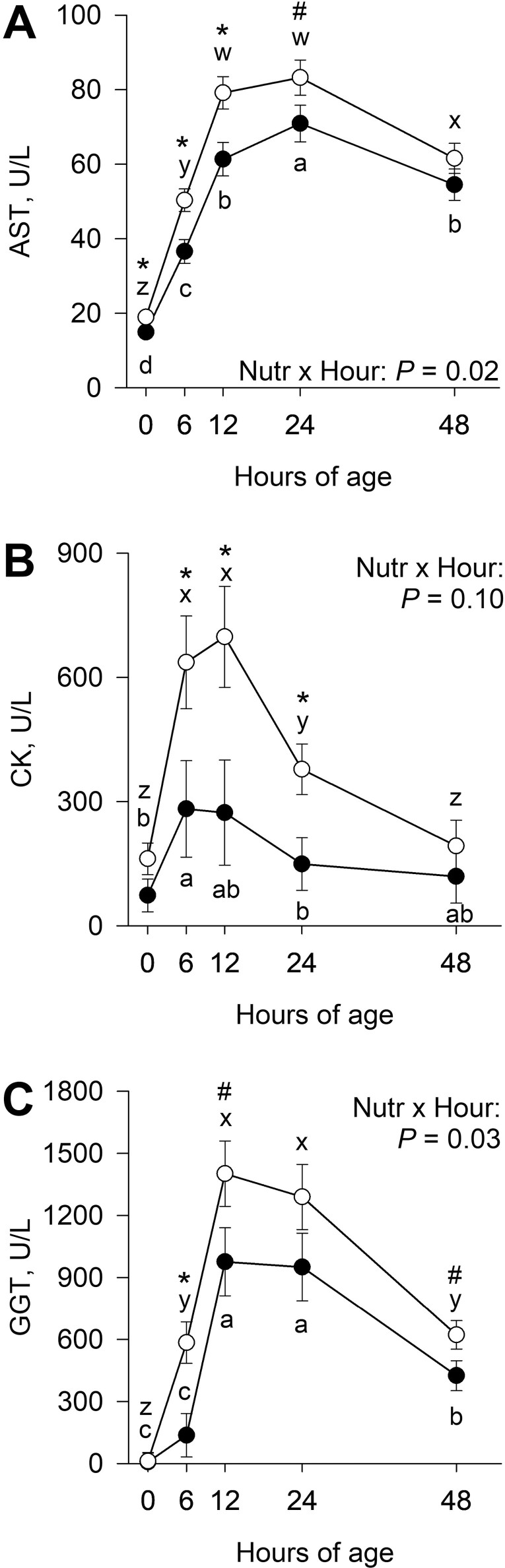
Effects of late gestational nutritional plane on neonatal calf serum aspartate aminotransferase (AST; Panel A), creatine kinase (CK; Panel B), and gamma-glutamyl transferase (GGT; Panel C). Calves were born to primiparous beef females individually-fed 100% (Control; *n* = 12; solid circles, ● ) or 70% (Nutrient Restricted; *n* = 13; open circles, ○ ) of metabolizable energy and metabolizable protein requirements for maintenance, pregnancy, and growth from day 160 of gestation to parturition. Least squares means ± SEM are presented. *Nutritional plane means within hour differ (*P* ≤ 0.05). #Nutritional plane means within an hour tend to differ (0.05 < *P* ≤ 0.10). ^a,b,c,d^Means differ (*P* ≤ 0.05) for Control calves across hours. ^w,x,y,z^Means differ (*P* ≤ 0.05) for Nutrient Restricted calves across hours.

There was an interaction (*P* ≤ 0.03) of late gestational nutritional plane × hour for serum AST and GGT activities and a tendency for an interaction (*P* = 0.10) of late gestational nutritional plane × hour for serum CK activity (Figure 5). Calves born to NR dams had greater (*P* ≤ 0.01) AST at 0, 6, and 12 h and tended to have greater (*P* = 0.07) AST at 24 h postnatal compared with CON. In calves born to CON dams, serum AST increased (*P* ≤ 0.01) from 0 to 24 h, then decreased (*P* < 0.001) from 24 to 48 h of age. Serum AST in NR calves increased (*P* < 0.001) between 0 and 12 h and then decreased (*P* < 0.001) from 24 to 48 h of age. Serum CK was greater (*P* ≤ 0.04) at 6, 12, and 24 h in calves born to NR dams. CK in calves born to CON dams increased (*P* = 0.02) from 0 to 6 h and tended to decrease (*P* = 0.09) from 12 to 24 h postnatal. In calves born to NR dams, CK increased (*P* < 0.001) from 0 to 6 h but then decreased (*P* ≤ 0.006) from 12 and 48 h of age. Serum GGT was greater (*P* = 0.006) at 6 h and tended to be greater (*P* ≤ 0.08) at 12 and 48 h of age in calves born to NR dams than CON. In calves born to CON dams, serum GGT increased (*P* < 0.001) from 6 to 12 h and then decreased (*P* < 0.001) from 24 to 48 h postnatal. In NR calves, serum GGT increased (*P* < 0.001) from 0 to 12 h and then decreased (*P* < 0.001) from 24 to 48 h of age.

**Figure 6.**
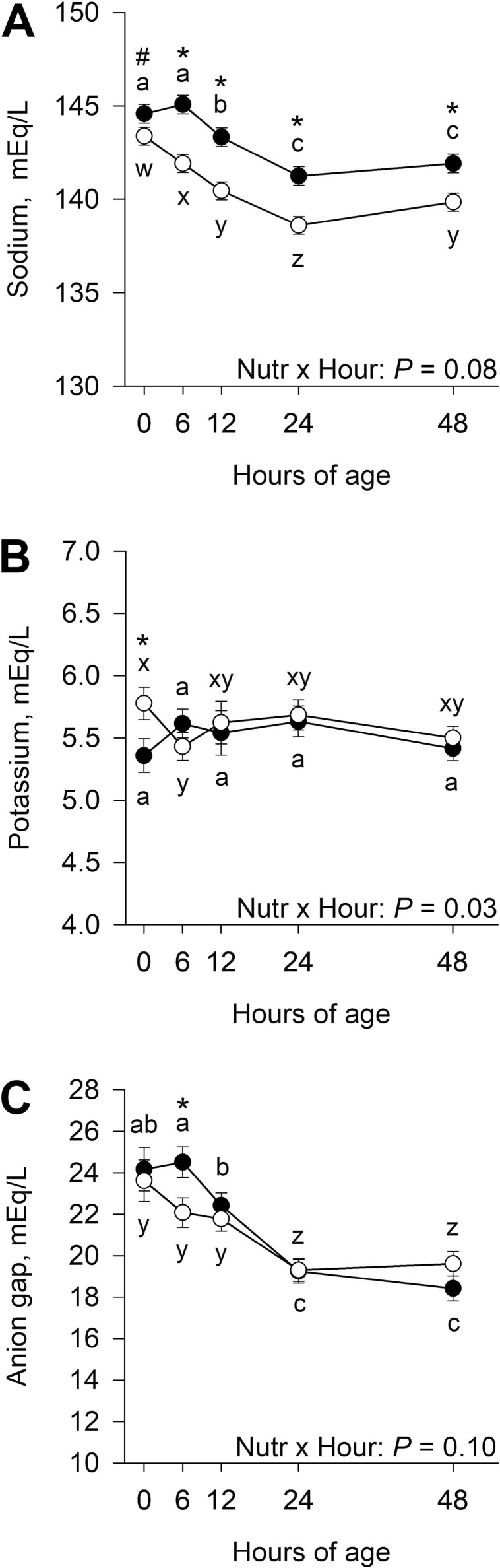
Effects of late gestational nutritional plane on neonatal calf serum sodium (panel A), potassium (panel B), and anion gap (panel C). Calves were born to primiparous beef females individually-fed 100% (Control; *n* = 12; solid circles, ● ) or 70% (Nutrient Restricted; *n* = 13; open circles, ○ ) of metabolizable energy and metabolizable protein requirements for maintenance, pregnancy, and growth from day 160 of gestation to parturition. Least squares means ± SEM are presented. *Nutritional plane means within hour differ (*P* ≤ 0.05). #Nutritional plane means within an hour tend to differ (0.05 < *P* ≤ 0.10). ^a,b,c^Means differ (*P* ≤ 0.05) for Control calves across hours. ^w,x,y,z^Means differ (*P* ≤ 0.05) for Nutrient Restricted calves across hours.

There was a tendency for a late gestational nutritional plane × hour interaction (*P* = 0.08) for serum sodium (Figure 6A) and an interaction (*P* = 0.03) of late gestational nutritional plane × hour for serum potassium (Figure 6B). Calves born to CON dams tended to have greater (*P =* 0.09) serum sodium at 0 h and had greater (*P* ≤ 0.004) sodium from 6 to 48 h postnatal. Within calves born to CON dams, sodium concentration decreased (*P* < 0.001) from 6 to 24 h of age. Serum sodium of NR calves decreased (*P* ≤ 0.003) from 0 to 24 h and then increased (*P* = 0.01) from 24 to 48 h of age. Serum potassium was greater (*P* = 0.03) in calves born to NR dams at 0 h postnatal when compared with CON. Potassium concentrations in calves born to CON dams tended to increase (*P* = 0.06) from 0 to 6 h and tended to decrease (*P* = 0.08) from 24 to 48 h. There was a decrease (*P* = 0.01) in serum potassium from 0 to 6 h of age in calves born to NR dams. There was no effect (*P* = 0.21) of the late gestational nutritional plane × hour interaction for serum chloride, but there tended to be an effect of nutritional plane (*P* = 0.08), where calves born to CON dams had greater concentrations compared with NR (100.6 ± 0.5 vs. 99.3 ± 0.5). There was no effect (*P* ≥ 0.12) of the late gestational nutritional plane × hour interaction or nutritional plane on serum calcium, phosphorus, or magnesium (data not shown). There tended to be an interaction (*P* = 0.10) of late gestational nutritional plane × hour for serum anion gap (Figure 6C). Calves born to CON dams had greater (*P* = 0.02) anion gap at 6 h of age compared with NR. Anion gap decreased (*P* ≤ 0.008) from 6 to 24 h in CON calves and decreased (*P* < 0.001) from 12 to 24 h in NR calves. There was no effect (*P* ≥ 0.46) of the late gestational nutritional plane × hour interaction or nutritional plane on neonatal calf serum bicarbonate concentrations (data not shown).

There was no effect (*P* ≥ 0.24) of the late gestational nutritional plane × hour interaction or nutritional plane on neonatal calf serum total or direct bilirubin (data not shown).

### Hematology

**Figure 7.**
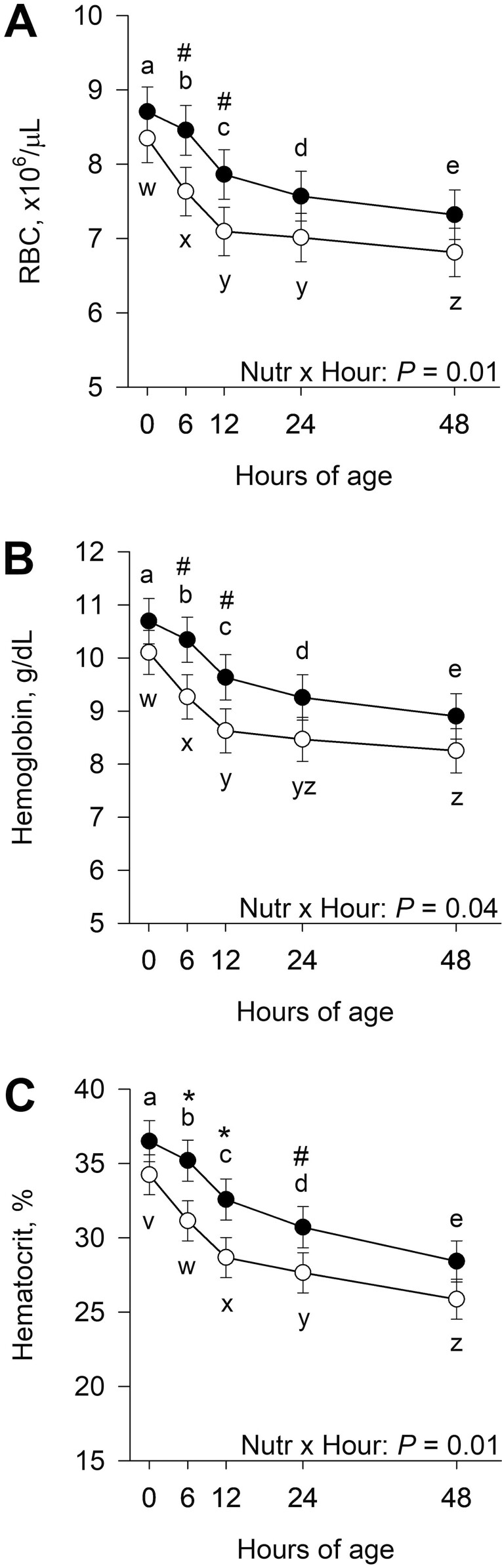
Effects of late gestational nutritional plane on neonatal calf red blood cell count (RBC; panel A), hemoglobin (panel B), and hematocrit (panel C). Calves were born to primiparous beef females individually-fed 100% (Control; *n* = 11 to 12; solid circles, ● ) or 70% (Nutrient Restricted; *n* = 13; open circles, ○ ) of metabolizable energy and metabolizable protein requirements for maintenance, pregnancy, and growth from day 160 of gestation to parturition. Least squares means ± SEM are presented. *Nutritional plane means within hour differ (*P* ≤ 0.05). #Nutritional plane means within an hour tend to differ (0.05 < *P* ≤ 0.10). ^a,b,c,d,e^Means differ (*P* ≤ 0.05) for Control calves across hours. ^v,w,x,y,z^Means differ (*P* ≤ 0.05) for nutrient restricted calves across hours.

There was an interaction (*P* ≤ 0.04) of late gestational nutritional plane × hour for RBC, hemoglobin, and hematocrit (Figure 7). Calves born to CON dams tended to have greater (*P* ≤ 0.09) RBC and hemoglobin at 6 and 12 h postnatal compared with NR. Red blood cells decreased (*P* ≤ 0.02) from 0 to 48 h postnatal in CON. In calves born to NR dams, RBC decreased (*P* ≤ 0.04) from 0 to 12 h and then from 24 to 48 h of age. Hemoglobin followed a similar pattern where it decreased (*P* ≤ 0.007) from 0 to 48 h of age in CON, and it decreased (*P* < 0.001) from 0 to 12 h and tended to decrease (*P* = 0.08) from 24 to 48 h in NR. Hematocrit concentrations were greater (*P* ≤ 0.04) at 6 and 12 h and tended to be greater (*P =* 0.10) at 24 h of age in calves born to CON dams compared with NR. In both CON and NR calves, hematocrit decreased (*P* ≤ 0.008) from 0 to 48 h postnatal. Neither the late gestational nutritional plane × hour interaction nor nutritional plane affected neonatal calf WBC (*P* ≥ 0.29), MCV (*P* ≥ 0.12), MCH (*P* ≥ 0.25), MCHC (*P* ≥ 0.14), or platelet count (*P* ≥ 0.70; Supplementary Figure S4).

## Discussion

Although the early neonatal period is the most likely time for pre-weaning beef calf death loss (USDA-APHIS, 2010), it is an under-studied period for impacts of poor maternal nutrition. We have previously reported that nutrient restricted heifers partitioned nutrients toward their fetuses rather than their own growth and maintenance of body condition, which conserved fetal growth in the current study (Redifer et al., 2023). Although calf size at birth was not affected, data reported here suggest that developmental or other differences likely occurred to affect calf vigor and physiology. Overall, calves born to nutrient restricted females in the current study were less vigorous at birth, showed more evidence of birth trauma, and had less blood oxygen carrying capacity. Conversely, they had greater serum Ig and minimally affected energy-related metabolites compared with calves born to control dams. Effects of late gestational nutrient restriction on neonatal calves observed in this study, or the lack thereof, were likely caused by a combination of the following factors: 1) fetal development, 2) calving difficulty and duration, 3) colostrum production, and 4) environment at calving and during the neonatal period.

Fetal development differences have been demonstrated repeatedly due to maternal nutrient restriction in ruminants (Caton and Hess, 2010; Meyer and Caton, 2016; Greenwood and Bell, 2019). Neonates are not only products of their fetal environment, but they are also greatly impacted by events of parturition (Arnott et al., 2012). The percentages of heifers assisted at calving and with fetal malpresentations at birth were not different, but 4 of 13 nutrient restricted dams were assisted at calving and 3 of 13 had fetal malpresentations (vs. 2 of 12 and 0 of 12, respectively, for control; Redifer et al. 2023). The current study was powered to have appropriate n for continuous and categorical data collected, but not for binomial data such as these. Previously, nutrient restriction during pregnancy has reduced pelvic growth in heifers (Bellows and Short, 1978; Kroker and Cummins, 1979), tended to increase duration of stage 2 parturition (Kroker and Cummins, 1979), and been observed to cause maternal fatigue or decreased effort during calving (Tudor, 1972; Kroker and Cummins, 1979). We postulate that all of these factors may have also caused more calving difficulty and trauma to calves born to nutrient restricted dams in the current study, regardless of whether visible dystocia or calving assistance occurred.

Neonatal metabolism is also affected by nutrient availability and ambient conditions postnatally. Despite 40% less total colostrum yield in nutrient restricted females in the current study, only total lactose was reduced, while total IgG, IgA, protein, and triglyceride yields were conserved compared with controls (Redifer et al., 2023). Additionally, fall-calving was used in the current study, and the average date of calving and ambient temperatures at birth were similar, allowing calves from both nutritional planes to generally benefit from thermoneutral conditions. Fall-calving allowed for less stress associated with cold, wet conditions altering the early life environment as we have observed previously in spring-born calves (Wichman et al., 2022).

### Vigor at birth

Using multiple measures, calves born to nutrient restricted dams were less vigorous in the current study. Kroker and Cummins (1979) reported similar findings where calves born to heifers fed a low nutritional plane during late gestation took longer to stand and to suckle. Other studies have reported no difference in calf vigor due to late gestational nutrition of beef cows or heifers (Anthony et al., 1986; Houghton et al., 1990; Wiley et al., 1991; Summers et al., 2015; Kennedy et al., 2019), but all experiments except that of Kroker and Cummins (1979) and Kennedy et al. (2019) used subjective calf vigor scores classifying calves using terms like “very vigorous” or “weak”. These subjective scores without clear definitions may not have been adequate to capture vigor differences, unlike the behavioral latencies or scores based on behaviors used in the current study.

Previous studies have associated reduced neonatal calf vigor with dystocia at birth (Hickson et al., 2008; Barrier et al., 2012; Vannucchi et al., 2015), which can be caused by negative effects on both neonatal physiology (Arnott et al., 2012; Mutinati et al., 2014) and maternal behavior (Dwyer, 2014). Rapid intervention when calving difficulty and fetal malpresentation were observed in the current study may have limited additional fetal stress from prolonged births (Murray and Leslie, 2013), as after observing fetal feet, duration of parturition was not affected by nutritional plane and was not correlated with any vigor measure (data not shown). Length of prior stages of parturition is unknown, however. Although calving difficulty likely affected calf vigor in this study, removing calves with assisted births did not change trends in the data, where the time to attempt to stand was 50% longer (*P* = 0.16), and time to stand was 60% longer (*P* = 0.09), for calves born to nutrient restricted dams. This suggests that other underlying impacts of late gestational nutrient restriction also decreased calf vigor at birth, such as fetal development or calving difficulty that did not result in assistance.

Kroker and Cummins (1979) reported that calf birth weight followed maternal nutritional plane, which may have contributed to poor vigor in calves born to heifers fed a low plane of nutrition. Although Dwyer et al. (2003) observed no effect of nutrient restriction during pregnancy on ­neonatal lamb vigor behaviors, they reported that nutrient restriction reduced birth weight, and smaller lambs took longer to display vigor behaviors regardless of maternal nutrition. Because fetal growth was not affected in the current study (Redifer et al., 2023), the reduced vigor of calves born to nutrient restricted dams is not likely related to calf size but may have been due to fetal development instead. Nutrient restriction during late gestation may have reduced fetal neurodevelopment, leading to impairment of complex movement coordination as previously suggested in Dwyer et al. (2003). In other species, experimentally-induced intrauterine growth restriction has been reported to result in brain network rearrangement in rabbits (Illa et al., 2017), reduced neuron numbers in guinea pigs (Mallard et al., 2000), and impairment of coordination for neurobehaviors in rats (Gramsbergen and Westerga, 1992).

Beef calves are often born into difficult environments and exposed to perinatal stressors (Arnott et al., 2012) that may limit vigor. Ultimately, neonatal vigor is most important to allow for consumption of colostrum shortly after birth. Although time to suckle was not measured due to pre-suckling blood and colostrum sampling in this study, we have previously reported that times to stand and suckle were positively correlated in beef calves (Wichman et al., 2019). Homerosky et al. (2017b) observed that the calves who did not consume colostrum within 4 h of birth were less likely to have optimal passive transfer of immunity and more likely to experience pre-weaning morbidity. Fall-born calves are faster to stand than those born in the spring (Wichman et al., 2022), so being born into a colder environment would have likely reduced vigor for all calves and may have exacerbated treatment differences. Overall, vigor differences observed here due to late gestational nutrient restriction may decrease calf health and survival in a production setting.

### Transfer of passive immunity

Calves born to nutrient restricted dams had greater indices of passive transfer (serum IgG, IgA, total protein, globulin, and GGT) in this study. All calves in both nutritional planes had serum IgG concentrations greater than the 8 to 9 mg/mL threshold suggested by Todd et al. (2018) and the 16 mg/mL threshold given by Wittum and Perino (1995). Using a 24 mg/mL IgG threshold (Dewell et al., 2006; Waldner and Rosengren, 2009), 5 calves born to control dams did not have optimal passive transfer. More calves could be classified as inadequate using serum total protein cutoffs of 5.0 g/dL (Weaver et al., 2000) or 5.6 g/dL (Todd et al., 2018), as shown in Figure 2, although this disparity between classifications using IgG and total protein has been reported previously (Todd et al., 2018). No neonatal calf morbidity was observed during the first 48 h of age, and no pre-weaning mortality occurred related to infectious disease. Pre-weaning, 1 calf (control) was administered electrolytes for scours, and 4 calves (2 control and 2 nutrient restricted) were treated with antibiotics for either navel infections or fevers (rectal temperature greater than 40.5 °C).

Nutrient restriction during pregnancy has often decreased or not affected calf serum Ig concentrations (reviewed by McGee and Earley, 2019), but similar results to the current study have been observed previously in calves born to dams after protein restriction or in poor BCS (Odde, 1988; Waldner and Rosengren, 2009). In the current study, greater serum IgG and IgA for calves born to nutrient restricted dams were likely due to colostrum from these heifers having 68% greater IgG and 72% greater IgA concentrations, despite having no difference in total IgG or IgA content (Redifer et al., 2023). More concentrated colostral Ig allowed for these calves to consume more Ig during their initial suckling events, while small intestinal Ig absorption was at its greatest (Matte et al., 1982). It has previously been reported that lambs born to nutrient restricted ewes and fed artificial colostrum to BW had greater 24-h serum IgG concentrations, which authors hypothesized was due to improved neonatal Ig absorption through increased transport or delayed small intestinal maturation (Hammer et al., 2011). Fetal and neonatal small intestinal development is affected by maternal nutrition (Meyer and Caton, 2016) and intrauterine growth restriction may increase small intestinal absorption of macromolecules in neonates (Sangild, 2003); thus, small intestinal differences may have also contributed to the current study. Assistance at calving has resulted in lower serum Ig concentrations (Waldner and Rosengren, 2009), but the greater incidence of calving assistance for nutrient restricted dams in this study does not appear to have negatively affected transfer of passive immunity.

Colostrum contains high GGT concentrations; therefore, neonatal serum GGT concentrations will sharply increase following colostrum intake (Knowles et al., 2000; Maden et al., 2003). Some studies suggest that serum GGT can be used to indicate successful passive transfer in both lambs (Maden et al., 2003) and calves (Perino et al., 1993), but serum IgG and GGT were not correlated in the current study. Because nutrient restricted dams had reduced colostrum yield, circulating GGT in their calves suggests that they had more concentrated GGT in their colostrum. The lack of IgG and GGT correlation in 48-h serum indicates that a poor relationship of colostral IgG and GGT existed or that 48 h was too late to observe the expected relationship in serum.

### Thermoregulation and metabolism

Following birth, thermogenesis is vital in preventing hypothermia as neonates transition to the extrauterine environment (Carstens, 1994; Danijela, 2015). Calves born to nutrient restricted dams had a lower rectal temperature at 0 h, but greater rectal temperature at 24 h. Using 15°C as the lower critical temperature and 25 °C as the upper critical temperature (NASEM, 2021), 2 control and 3 nutrient restricted calves were born in ambient temperatures below this range, whereas 2 control and 4 nutrient restricted calves were born in ambient temperatures above this range. The similar mean ambient temperatures at birth for calves from both nutritional planes suggest that the 0 h difference was not due to ambient temperature. Rectal temperature at 0 h was negatively correlated with age at measurement (*r* = −0.53, *P* = 0.006). Because 0 h data were obtained after calves successfully stood, nutrient restricted calves were 15 min older (29.4 ± 4.2 vs. 44.4 ± 4.2 min; *P* = 0.02) than control calves at the 0 h sampling. Vannucchi et al. (2019) reported that Holstein calves in relatively thermoneutral environments lost approximately 0.4 °C from 30 to 50 min of age; thus, the rectal temperature difference observed pre-suckling may have been solely due to age in the current study. Although dystocia can decrease neonatal calf rectal temperature (Barrier et al., 2013), similar results were observed when assisted calvings were removed (data not shown). Previously, Carstens et al. (1987) observed no difference in calf rectal temperature at birth, but 11.4% less heat production between 5 and 13 h postnatal in calves born to protein restricted dams. Martin et al. (1997) reported no differences in metabolic rate at 37°C or brown adipose tissue mass or function due to maternal protein restriction in another study. We have previously shown that fall-born calves have more stable rectal temperatures than those born in the spring (Wichman et al., 2022); therefore, thermogenesis and rectal temperatures may have been affected differently by late gestational nutrient restriction in colder conditions requiring greater thermogenesis.

Neonatal calf energy metabolism is critical during the transition from parenteral to enteral nutrition (Hammon et al., 2012), especially given the thermoregulation challenges that often exist (Danijela, 2015). Calves are born with limited glycogen that they mobilize quickly after birth, while also increasing gluconeogenesis to make up for the glucose deficit caused by limited lactose intake from colostrum (Hammon et al., 2013). During this time, fat mobilization also increases to provide energy (Hammon et al., 2012), and an increase in circulating NEFA is observable by 6 h of age in calves born into both cold and warm environments (Wichman et al., 2022), which provides an additional non-glucose substrate for brown adipose tissue (Carstens, 1994). Digestion and absorption of colostral lipids increase circulating triglycerides by 6 h of age (Larson-Peine et al., 2022), but can be variable, likely based on fat concentration of colostrum (Duncan et al., 2023). Finally, amino acids can also be deaminated to provide carbon skeletons for gluconeogenesis (Hammon et al., 2013), resulting in elevated circulating urea N.

Taken together, the circulating glucose, NEFA, triglycerides, urea N, and insulin of calves in the current study do not show marked contrasts in energy metabolism due to late gestational nutrient restriction. Given that fetal growth was not affected (Redifer et al., 2023) and ambient conditions were not harsh, this lack of differences in energy metabolism likely demonstrates calves from both late gestational nutritional planes had adequate available energy sources in their early life environments. In this study, colostrum triglyceride concentration and yield were not affected by nutrient restriction, and although free glucose was greater in colostrum from control dams, the concentration and total yield of free glucose were too low to provide a major glucose source (Redifer et al., 2023). Furthermore, protein concentration was decreased by nutrient restriction (Redifer et al., 2023), but it is unknown how much of this was due to casein differences rather than Ig. Total lactose available in colostrum was greater for control calves, but the concentration was not (Redifer et al., 2023); thus, lactose consumption was not affected unless meal size differed between nutritional planes. Colostrum yield was driven by total lactose (Linzell and Peaker, 1971); thus, lower lactose production by nutrient restricted dams decreased total colostrum available for calves during a meal. Voluntary colostrum consumption was not measured in this study, but as much as 2.4 kg of colostrum consumption would have been possible on average (up to 8% of BW within first 9 to 12 h of life; McGee and Earley, 2019). Only an average of 1.1 kg and 0.7 kg would have been available for control and nutrient restricted calves, respectively, assuming the single rear quarter sampled accounted for 31.2% of total colostrum (Rathert-Williams et al., 2023). Milk yields were not measured in the current study until day 21 of lactation, but we hypothesize that available transition milk was also less for calves born to nutrient restricted dams. Therefore, calves born to nutrient restricted dams may have been more limited in their total nutrients available during the neonatal period due to decreased colostrum and transition milk yields.

Colostrum urea N was less for nutrient restricted dams, expressed both as concentration and total content (Redifer et al., 2023). Given that less urea N was consumed by their neonatal calves without ruminal microbes to utilize it, we hypothesize that similar serum urea N (and an extended initial increase in serum urea N) among treatments indicates more amino acids were being deaminated by calves born to nutrient restricted females. These deaminated amino acids may have provided enough energy substrates for calves from nutrient restricted dams to maintain their glucose status without elevated NEFA. Insulin concentrations in calves born to first-parity dams were greater compared with the current study, but they showed a lack of change over the first 72 h of life similar to the current study (Duncan et al., 2023). Cold stress of these calves was minimal due to ambient temperature, bedding provided, and shelter from precipitation and wind. Thus, calves in this study likely were able to maintain thermogenesis and glucose status through different metabolic approaches even with differences in colostrum intake. If these calves had experienced cold stress, we hypothesize that calves born to nutrient restricted dams would have had decreased circulating glucose and increased NEFA, as observed in calves born to primiparous dams by Duncan et al. (2023), given they had less colostrum available.

Previous studies reported that maternal nutrient restriction in beef (Long et al., 2021) and dairy × beef (Prior and Scott, 1977) had no effect on fetal plasma glucose concentrations or fetal liver gluconeogenic activity, which suggests glucose supply to meet fetal demands is prioritized over maternal demands (Hammon et al., 2012). Results from the current study are in agreement with the aforementioned studies and support this notion of prioritization of fetal demands as there was no effect of maternal treatment on neonatal energy metabolites.

### Indicators of stress and trauma

Changes in calf blood chemistry over time presented here are similar to our previous observations for healthy, fall-born neonatal calves (Larson-Peine et al., 2022). Few blood chemistry or hematology measures were divergent at 0 h; thus, the difference in age at the pre-suckling sampling caused by vigor differences may not have influenced data interpretation.

Neonatal calf AST, CK, and creatinine were greater for calves born to nutrient restricted dams within the first 24 h of life, and have been associated with trauma (tissue damage, edema, injury) of difficult births. Both AST and CK are important enzymes for amino acid and energy metabolism, respectively, that are commonly found in liver and muscle cells; however, when muscle damage or inflammation occurs these enzymes are released into circulation causing increased concentrations (Russell and Roussel, 2007; Pearson et al., 2019). Pearson et al. (2019) observed greater AST and CK concentrations in calves whose births were considered a difficult assist compared with unassisted or easy assists. These authors also reported that calves with poor vitality indicators such as pale mucous membranes, incomplete tongue withdrawal, and weak suckling reflex had greater AST and CK concentrations (Pearson et al., 2019). Therefore, these observations are in good agreement with the reduced neonatal vigor that was displayed by calves born to nutrient restricted dams. Serum creatinine may indicate greater trauma during calving (Perrone et al., 1992), also suggesting more difficult parturition following nutrient restriction. Creatinine concentrations can also be influenced by muscle mass (Russell and Roussel, 2007), but as there were no differences in calf size or shape measures at birth (Redifer et al., 2023), this is unlikely the cause of the difference observed in the current study.

Although CK differences lessened when calves with assisted births were removed from the dataset, AST and creatinine differences persisted (data not shown). Circulating cortisol was not affected by nutrient restriction, but this may be due to the sampling times used in the current study, as we have previously suggested (Duncan et al., 2023). We have reported similar observations where calves born to first-parity dams had greater AST, CK, and creatinine despite similar cortisol compared with calves born to multiparous females, even though there was limited incidence of calving assistance (Duncan et al., 2023). Moreover, spring-born neonatal calves had greater AST and CK than fall-born calves, following vigor data, in another study from our lab in which calf data were removed if calving assistance was necessary (Wichman et al., 2022). Overall, calves born to nutrient restricted dams in the current study appear to have experienced more stressful calving and early life experiences, regardless of visible dystocia.

Anion gap is typically used to evaluate metabolic acidosis (Russell and Roussel, 2007), which can depress vigor and muscle coordination, leading to increased calf mortality (Vannucchi et al., 2015; Homerosky et al., 2017b). The greater anion gap at 6 h in calves born to control heifers is likely not due to metabolic acidosis, given they were more vigorous, but may have been caused by elevated sodium concentrations at that sample time point. Sodium concentrations are greatest in colostrum and decrease with production of milk (McGrath et al., 2016); thus, sodium intake of calves born to nutrient restricted dams may have been reduced by colostrum yield differences. Concentrations of chloride often follow the pattern of sodium, because renal reabsorption of both occurs simultaneously (Russell and Roussel, 2007). Concentrations of albumin are reflective of hydration status (Russell and Roussel, 2007), so serum albumin concentrations suggest that hydration status of calves did not differ. Overall, electrolyte status was not greatly affected by maternal nutrient restriction in the current study.

Red blood cells, hemoglobin, and hematocrit were not affected at birth but were less in calves born to nutrient restricted dams from 6 to 24 h. The general decrease in RBC measures observed after birth has been observed previously (Kurz and Willett, 1991; Egli and Blum, 1998; Knowles et al., 2000), and it has been suggested that delayed responses to hemorrhage during birth may be the cause (Homerosky et al., 2017a). Calves born with assistance due to dystocia had less RBC or hematocrit within the first day of life in other studies (Egli and Blum, 1998; Homerosky et al., 2017a). Hematocrit has also generally been lower on the first day of life for calves with meconium staining, pale mucous membranes, incomplete tongue withdrawal, and weak suckling reflex (Homerosky et al., 2017a). This suggests that the lower blood oxygen-carrying capacity in nutrient restricted calves was a result of more trauma experienced during birth. These trends became less pronounced when calves with assisted births were removed, but hematocrit results persisted (data not shown). Kume et al. (1998) also observed that feed restriction of pregnant dairy females resulted in neonatal calves with less hematocrit and hemoglobin without altered birth weight; thus, our observations may not have been influenced only by calving assistance or dystocia. Elevated RBC, hemoglobin, and hematocrit can be caused by a decrease in plasma volume or following a splenic contraction (Roland et al., 2014). An accompanying increase in total protein or albumin was not observed concurrently in calves born to control dams, which excludes dehydration or a decrease in plasma volume as the cause of greater RBC in these calves. Bilirubin was not affected by late gestational nutritional plane, also suggesting that differences in RBC breakdown did not cause these observations.

Limited neonatal beef calf hematology data from 0 to 48 h of age are available for comparison, so our data may provide a useful resource for values in very young calves. Hematology changes over time were less pronounced than for most blood chemistry values and had similar patterns to our previous observations in neonatal foals (Duncan et al., 2020). All RBC, hemoglobin, and MCH values from the current study were within the 90% confidence intervals of the reference intervals proposed by Panousis et al. (2018) using calves from 1 to 9 d old. Hematocrit (3 above), WBC (1 below, 2 above), and platelet (3 below) values were predominantly within these reference intervals (Panousis et al., 2018), with all outside values from 0, 6, or 12 h sampling. Neonatal calf MCV (20% above) and MCHC (23% below) were less likely to be within these reference intervals (Panousis et al., 2018).

## Conclusion

Although no differences were observed in fetal growth in the current study, calves born to nutrient restricted dams were less vigorous after birth and showed more signs of trauma at calving. Despite this, calves born to nutrient restricted dams had successful transfer of passive immunity and minimal differences in energy metabolism. The relatively thermoneutral environment and intensive management in the current study likely decreased overall calf metabolic stress by minimizing the need for thermogenesis; therefore, the effects of nutrient restriction may result in poor pre-weaning health and survival outcomes in many cow-calf production scenarios, especially those involving cold stress. Data from this study suggest that impaired transfer of passive immunity is not the major threat to calves born to nutrient restricted females. Overall, the current study (including previously-published and forthcoming data) allows for integration of many maternal and offspring effects caused by late gestational nutrient restriction, expanding our knowledge of developmental programming in beef cattle and giving greater insight into effects on the neonatal calf. Better understanding of the ramifications of poor maternal nutrition on neonatal beef calves can improve pre-weaning survival and productivity.

## Supplementary Material

skad342_suppl_Supplementary_Figures_1-4

## Abbreviations

ADF: acid detergent fiber
AST: aspartate aminotransferase
BCS: body condition score
BW: body weight
CK: creatine kinase
CP: crude protein
DM: dry matter
ELISA: enzyme linked immunosorbent assay
GGT: gamma-glutamyl transferase
Ig: immunoglobulin
MCH: mean corpuscular hemoglobin
MCHC: mean corpuscular hemoglobin concentration
MCV: mean corpuscular volume
ME: metabolizable energy
MP: metabolizable protein
NDF: neutral detergent fiber
NEFA: non-esterified fatty acids
RBC: red blood cells
RIA: radioimmunoassay
WBC: white blood cells
