# Supplementary material for: Maternal nutrient restriction during late gestation reduces vigor and alters blood chemistry and hematology in neonatal beef calves
Source: J Anim Sci. 2023 Oct 3;101:skad342. doi: 10.1093/jas/skad342 (PMC10648570; doi:10.1093/jas/skad342)
Supplement: skad342_suppl_Supplementary_Figures_1-4 [file skad342_suppl_supplementary_figures_1-4.docx]

**
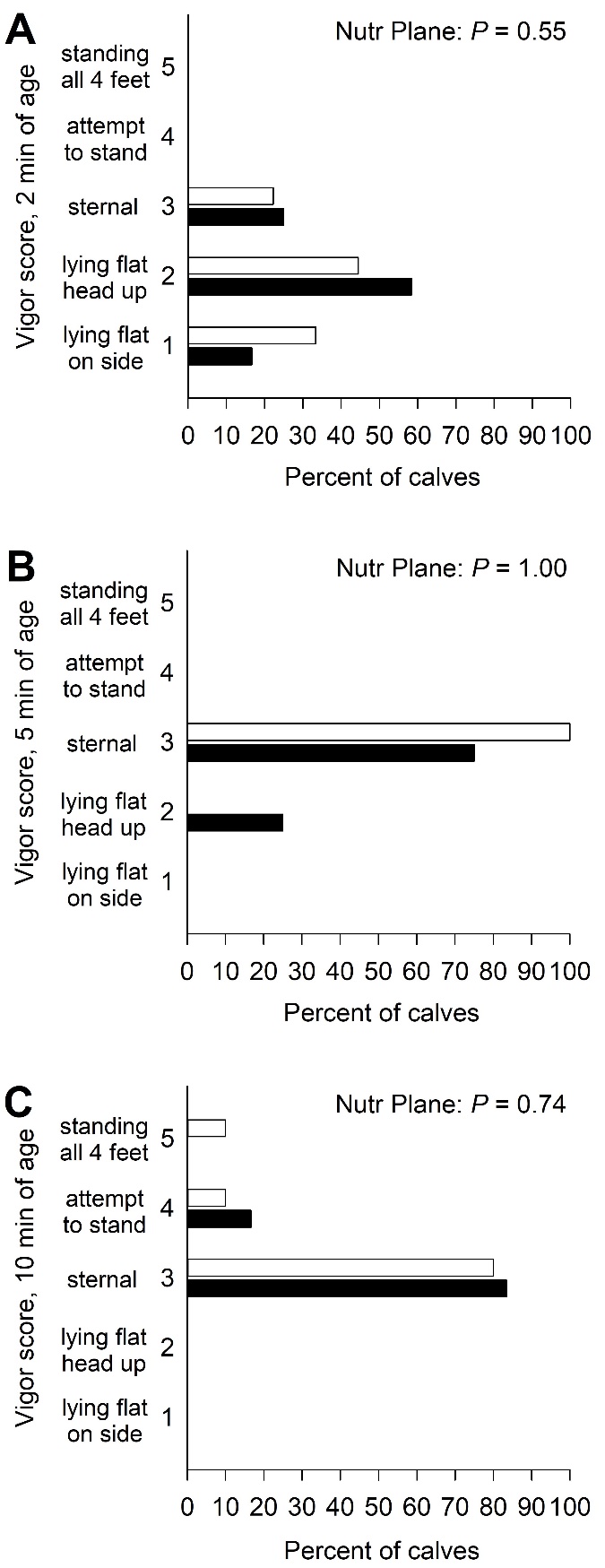
**

**Supplementary Figure 1.** Effects of late gestational nutritional plane on frequency of beef calf vigor score at 2 min (Panel A), 5 min (Panel B), and 10 min (Panel C) of age. Calves were born to primiparous beef females individually-fed 100% (Control; *n* = 12; solid bars, ■) or 70% (Nutrient Restricted; *n* = 8 to 13; open bars, □) of metabolizable energy and metabolizable protein requirements for maintenance, pregnancy, and growth from d 160 of gestation to parturition. Calves pulled in the chute were not included for these time points.


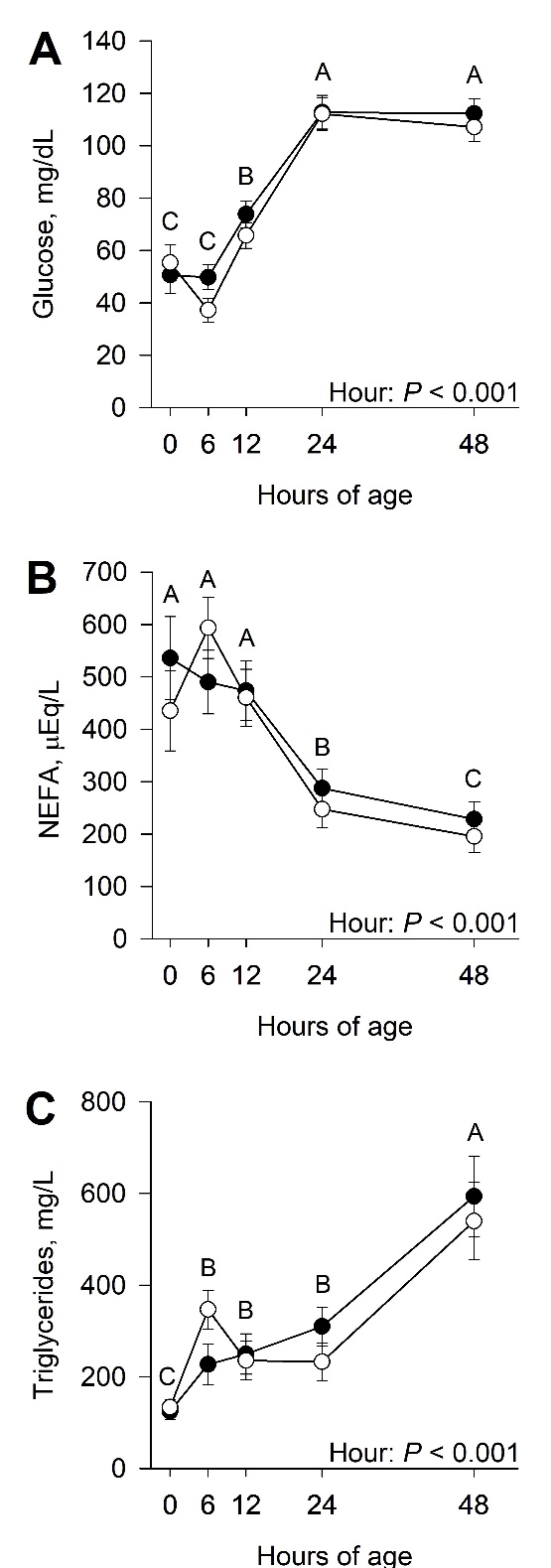


**Supplementary Figure 2.** Effects of late gestational nutritional plane on neonatal calf serum glucose (Panel A), serum non-esterified fatty acids (NEFA; Panel B), and plasma triglycerides (Panel C). Calves were born to primiparous beef females individually-fed 100% (Control; *n* = 12; solid circles, ●) or 70% (Nutrient Restricted; *n* = 13; open circles, ○) of metabolizable energy and metabolizable protein requirements for maintenance, pregnancy, and growth from d 160 of gestation to parturition. Least squares means ± SEM are presented. There was no effect of nutritional plane × hour or nutritional plane (*P* ≥ 0.18). ^ABC^ Means differ (*P* ≤ 0.05) across hours.


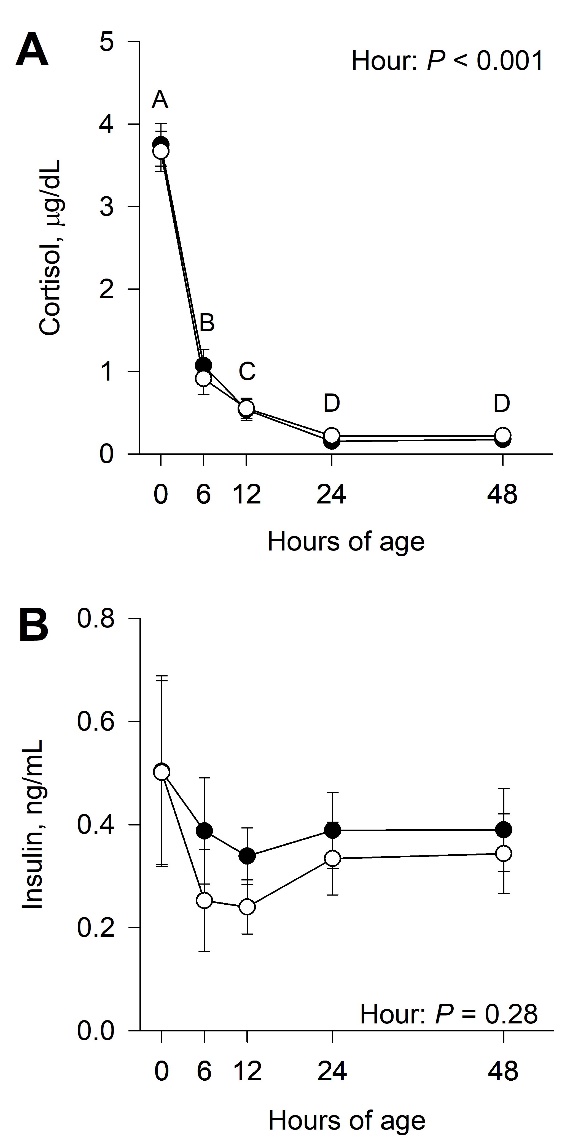


**Supplementary Figure 3**. Effects of late gestational nutritional plane on neonatal calf plasma cortisol (Panel A) and insulin (Panel B). Calves were born to primiparous beef females individually-fed 100% (Control; *n* = 12; solid circles, ●) or 70% (Nutrient Restricted; *n* = 13; open circles, ○) of metabolizable energy and metabolizable protein requirements for maintenance, pregnancy, and growth from d 160 of gestation to parturition. Least squares means ± SEM are presented. There was no effect of nutritional plane × hour or nutritional plane (*P* ≥ 0.46). ^ABCD^ Means differ (*P* ≤ 0.05) across hours.


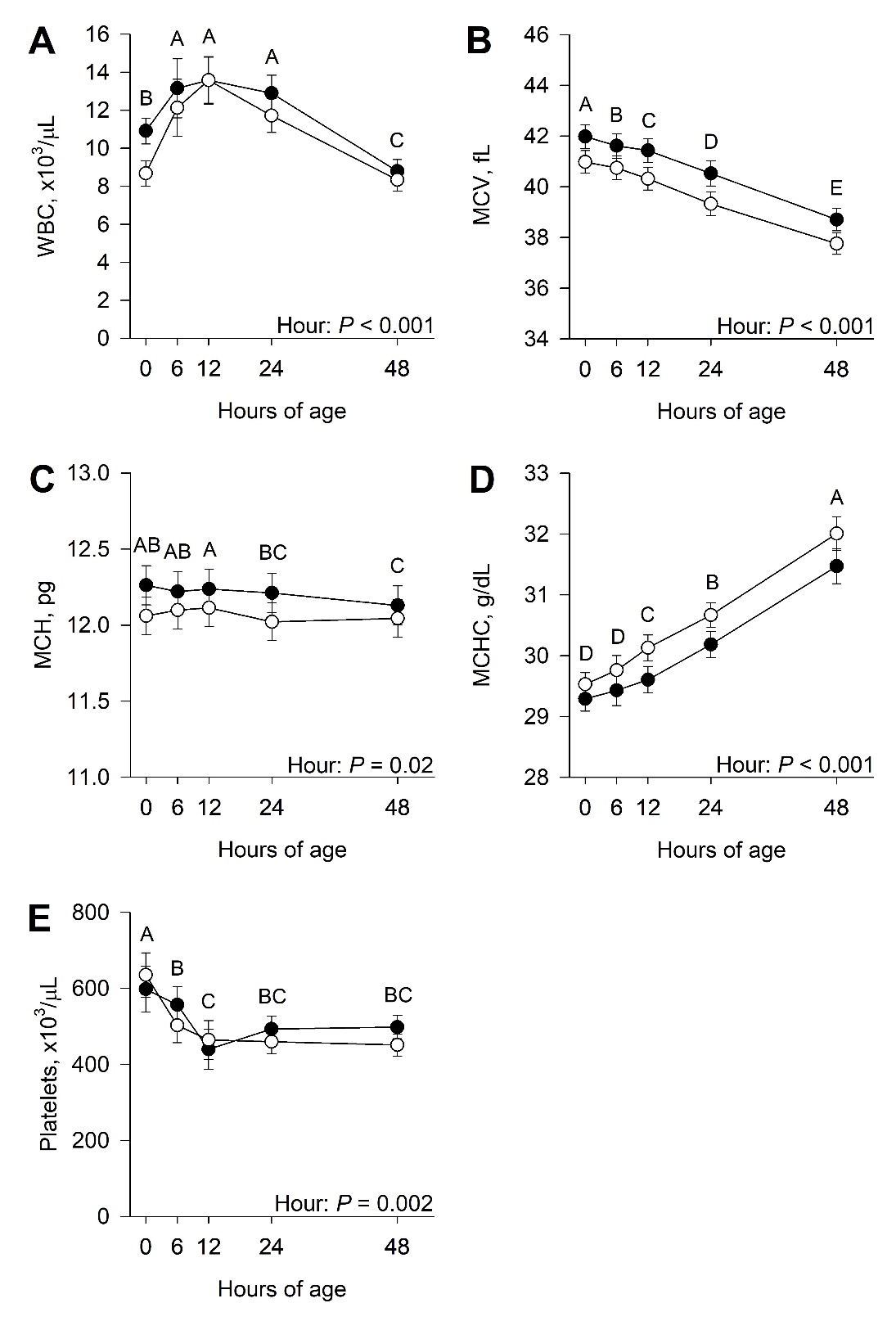


**Supplementary Figure 4.** Effects of late gestational nutritional plane on neonatal calf white blood cell count (WBC; Panel A), mean corpuscular volume (MCV; Panel B), mean corpuscular hemoglobin (MCH; Panel C), mean corpuscular hemoglobin concentration (MCHC; Panel D), and platelets (Panel E). Calves were born to primiparous beef females individually-fed 100% (Control; *n* = 11 to 12; solid circles, ●) or 70% (Nutrient Restricted; *n* = 13; open circles, ○) of metabolizable energy and metabolizable protein requirements for maintenance, pregnancy, and growth from d 160 of gestation to parturition. Least squares means ± SEM are presented. There was no effect of nutritional plane or nutritional plane × hour (*P* ≥ 0.12). ^ABCDE^ Means differ (*P* ≤ 0.05) across hours.
